# The Effect of Photodynamic Therapy on *Enterococcus* spp. and Its Application in Dentistry: A Scoping Review

**DOI:** 10.3390/pharmaceutics16060825

**Published:** 2024-06-18

**Authors:** Mariaignacia Rubilar-Huenchuman, Camilo Ortega-Villanueva, Iván A. González, Christian Erick Palavecino

**Affiliations:** 1 Laboratorio de Microbiología Celular, Facultad de Medicina y Ciencias de la Salud, Universidad Central de Chile, Lord Cochrane 418, Santiago 8330546, Chile; mariaignacia.rubilar@alumnos.ucentral.cl (M.R.-H.); camilo.ortega@alumnos.ucentral.cl (C.O.-V.); 2 Departamento de Química, Facultad de Ciencias Naturales, Matemática y del Medio Ambiente, Universidad Tecnológica Metropolitana, Las Palmeras 3360, Ñuñoa, Santiago 7800003, Chile; igonzalezp@utem.cl

**Keywords:** *Enterococcus* spp., multidrug-resistant, photodynamic therapy

## Abstract

*Enterococci* spp. are Gram-positive bacteria that cause mild to severe infections, many associated with the oral cavity, such as periapical infections and healthcare-associated infections (HAIs). Many of these infections become serious diseases that are difficult to resolve, specifically when multidrug-resistant (MDR) strains cause them. In recent years, the number of MDR strains of *Enterococcus* spp. has increased significantly. This increased prevalence of MDR strains produces significant pressure to generate more antimicrobial therapies, but there is a decline in the production of new antibiotics, driving the development of complementary therapies, such as photodynamic therapy (PDT). PDT combines a photosensitizer agent (PS), light, and oxygen to cause photooxidative stress in bacterial cells. PDT can eradicate *Enterococcus* spp. contaminations, improve the classic cleaning processes, and eradicate the bacteria in dental pieces. PDT’s effectiveness can be improved with nanoparticles that function as carriers. Our work aims to describe the advances in PDT against *Enterococcus* spp. as a complement to antibiotic therapy, focusing on infections by *Enterococcus faecium* and *Enterococcus faecalis*, dental hygiene, and using nanoparticles to improve the antimicrobial effect. A systematic bibliographic search without a meta-analysis was conducted on various databases, using inclusion and exclusion criteria to identify the most relevant research. Of the 193 non-redundant articles found, 65 were selected for a systematic review, from which a summary table was created and a manual description was made. Photodynamic therapy for treating *E. faecium* and *E. faecalis* is a widely studied area, with promising results concerning bactericidal effectiveness and reductions in biofilm formation, particularly in regard to dental hygiene. Because most of the studies were conducted in vitro or ex vivo, the results indicated that there were not sufficient data to initiate clinical trials for safety and efficacy studies on humans.

## 1. Introduction

The increasing prevalence of multidrug-resistant (MDR) bacteria is a severe public health problem and, in the opinion of the World Health Organization (WHO), an urgent global health problem [1]. Although antibacterial resistance arises naturally over time, recent decades have seen accelerated growth in humans, animals, and plants due to inappropriate or excessive use of antibiotics [2]. MDR bacteria cause nearly 700,000 deaths yearly due to failure to treat infections, which could lead to 10 million deaths in the next 25 years [3,4]. To focus efforts on stopping the progression of MDR bacteria, in 2017, the WHO published a global list of multidrug-resistant bacteria that deserve to be prioritized in terms of research into new antibiotic drugs [1]. *Enterococcus* spp. is one of them, and it is classified as high-risk in the priority-2 group including other ESKAPE bacteria [5]. Within this group, *Enterococcus* spp. stands out for being one of the members capable of precipitating intra-hospital epidemic outbreaks [6]. The *Enterococcus* spp. is a Gram-positive coccus, grouped in chains or pairs, and a facultative aerobe belonging to the *Enterococcaceae* family. Enterobacteria and *Enterococcus* spp. are common residents of the gastrointestinal tract as well as the oral cavity. Although *Enterococcus* spp. were initially classified as group D streptococci, nucleic acid hybridization studies grouped them into an individual genus [7].

The *Enterococcus* genus contains more than 50 species, and commensal gut enterococci are not typically pathogenic in healthy hosts. The most clinically relevant *Enterococcus* species is *Enterococcus faecalis* (80–90%), followed by *Enterococcus faecium* (10–15%), both of which mainly cause soft tissue infections such as abscesses, urinary tract infections (UTIs), and endocarditis [7,8,9]. Also, *E. faecalis* is strongly associated with apical periodontitis [10,11]. Interestingly, due to its resistance to antiseptics and disinfectants used in dental hygiene, *E. faecalis* is described as an essential cause of endodontic treatment failure, isolated in up to 77% of chronic and persistent periapical lesions cases [11]. In persistent intra-radicular *E. faecalis* infections, interaction with the host immune cells can lead to apical periodontitis that does not heal and can generate tissue destruction with bone resorption [12,13]. In this way, *E. faecalis* is considered the most significant species found in cases of duct systems with periradicular lesions [14,15]. For example, in one case, enterococci were the main Gram-negative facultative bacteria isolated from the subgingival microbiota, amounting to 7.4% of all samples, representing 9.8% in periodontitis and 7.8% in gingivitis compared to 2.2% in periodontal health [16]. Although the corresponding pathogenic mechanisms have not yet been wholly elucidated, *Enterococcus* can successfully withstand the resistance to colonization presented by host commensal bacteria; several virulence factors allow it to adhere to host tissues, invade, secrete toxic products, and produce biofilms [17]. Enterococcal biofilms are associated with various infections, including urinary tract infections, wounds, a dysbiotic gastrointestinal tract, and endocarditis. Enterococci also frequently cause opportunistic infections associated with biofilms [18]. For example, the operon encoding the endocarditis-biofilm-associated pilus (Ebp) was shown in vivo to be essential for producing catheter-associated UTIs and infective endocarditis [19].

*Enterococcus* spp. are characterized by growing in wide temperature ranges ranging from 10 °C to 45 °C and resisting unfavorable environmental conditions such as a pH of 9.6, desiccation, and the presence of 6.5% NaCl_2_. This resistance is aided by its ability to hydrolyze esculin in the presence of 40% bile and the production of pyrrolidonyl arylamidase (PYR) [7]. Furthermore, over 90% of *Enterococcus* strains express the Lancefield group D lipoteichoic antigen in their cell walls [9]. Such survival capacity, in addition to intrinsically resistant antibiotics such as aminoglycosides and β-lactam-based antibiotics, makes it one of the most prevalent producers of healthcare-associated infections (HAIs). Recent studies indicate that *Enterococcus* spp. and vancomycin-resistant *Enterococcus* (VRE) strains represent 10.9% and 1.1% of all pathogens isolated from HAI patients [20]. *Enterococcus* spp. manifest intrinsic resistance to multiple families of commonly used antibiotics, regardless of previous exposure or gene transfer, such as cephalosporins, synthetic penicillins, aminoglycosides, lincosamides, and streptogramins [21]. In 1950, vancomycin was introduced as the chosen antibiotic to treat *Enterococcus* resistant to aminoglycosides; currently, about 80% of *E. faecium* clinical isolates are vancomycin-resistant [7,8]. Therefore, the need arises to generate new drugs or alternative therapies to stop the advance of MDR *Enterococcus* spp., wherein photodynamic therapy (PDT) may help.

PDT is a technique wherein generated reactive oxygen species (ROS) produce oxidative stress that affects cellular organic structures such as the bacterial envelope [22,23,24]. ROS are produced by activating non-toxic dyes called photosensitizers (PSs), which absorb photon-quantized visible-light energy and transmit it to molecular oxygen in the environment [25]. The photodynamic process is induced by exciting an electron in the PS to a higher energy orbit, generating an excited state. This excited state is short-lived since the energy may be lost as fluorescence or gained by the surrounding molecular oxygen through an intersystem crossing process. The molecular oxygen gains the energy along with an electron, a process called the Type I effect, or alone, a process called the Type II effect, thereby producing ROS. An ROS produced via the Type I effect is called a superoxide radical (O_2_^−•^), which, in turn, produces hydrogen peroxide (H_2_O_2_) and hydroxyl free radicals (HO^•^). Conversely, the Type II effect transfers only energy to triplet-state molecular oxygen (^3^O_2_), forming singlet-state oxygen (^1^O_2_°) [25]. The cytotoxicity produced by PDT is mainly caused by oxidative stress induced by light (photooxidative stress), which causes irreparable damage to bacterial structures such as cell membranes and nucleic acid [8].

The effectiveness of PDT depends mainly on the light and PS dose employed, as well as the excitation time interval; when excited long enough, the generated quantity of ROS will be sufficient to cause bacterial cell death. Resistance is unlikely, as the PDT-derived ROS affect the bacterial structures in an unspecific manner. To date, there are no reports of microorganisms becoming resistant to PDT [26]. The primary PS light activation sources must show monochromaticity and have several wavelength options, such as light-emitting diodes (LED), gas discharge lamps, and lasers, which are the most used [27]. Light penetration into tissues will vary depending on the wavelength used; the longer the wavelength, the more penetrating the light. In contrast, the longer the wavelength, the less energetic it is. Tissues mainly absorb light of shorter wavelengths <650 nm, resulting in a higher skin photosensitivity risk. On the other hand, light of longer wavelengths (>850 nm) does not have enough energy to elevate PSs to a triplet state capable of transitioning oxygen molecules into an excited state, preventing the generation of ROS [28]. Generally, the most effective visible-light range for giving tissues enough energy to activate a photosensitizer is between 650 and 850 nm. However, the choice of photosensitizer and light source will also depend on the intended application. For example, photosensitizers activated by blue light (<500 nm) are useful for treating superficial skin or oral cavity infections [29]. Also, PSs may be combined with carrier molecules such as nanoparticles, which may help to prevent several limitations, such as photobleaching, the need for proximity to the target, or low oxygen excitation [30]. Therefore, this work will help researchers and treating physicians choose various alternatives correctly. To produce a valid and updated document, we carried out a systematic bibliographic search that will help readers identify the current stage of photodynamic therapy against *Enterococcus* spp. and where future efforts should focus.

## 2. Materials and Methods

Databases such as PubMed, Scopus (Elsevier), and Google Scholar were searched for a scoping review without meta-analysis. The methodology included the use of the following search keywords: *Enterococcus faecium*, *Enterococcus faecalis*, photodynamic therapy, photosensitizer, clinical isolates, nanoparticles, and antibiotic resistance. Following the PRISMA 2020 guidelines, this article’s relevance was evaluated using inclusion and exclusion criteria. Inclusion criteria included the following: articles published between 2011 and 2023; PDT for treating ESKAPE group bacteria including *Enterococcus faecium* or *Enterococcus faecalis*; PDT studies on *Enterococcus* spp., *Enterococcus faecium*, or *Enterococcus faecalis* strains; in vitro or in vivo PDT studies; and PDT studies involving typo strains such as those from the ATCC (American Type Culture Collection) or the use of clinical isolates. Exclusion criteria excluded articles published before 2011; articles to which we did not have access; studies that do not declare photodynamic information such as the photosensitizer and wavelength used, the energy applied, and the quantification of the reduction in microbial load; articles focused on bacteria from the ESKAPE group but omitting *Enterococcus faecium* or *Enterococcus faecalis*; and articles not published in scientific journals or that lacked clinical significance.

## 3. Results

### 3.1. Data Extraction Scheme and Article Selection

**Figure 1:**
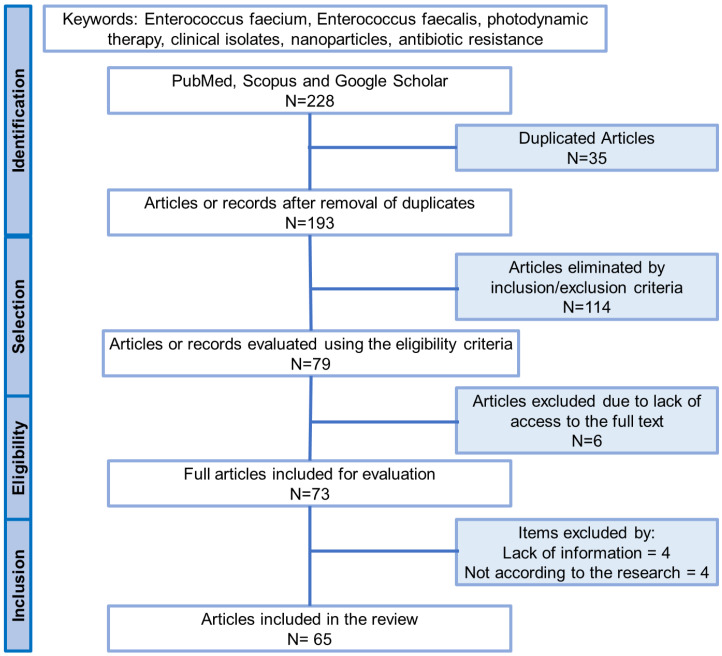
Data extraction according to the PRISMA guidelines.

As shown in Figure 1, the number of articles found by each researcher in the first instance was 228. Of these, 35 were redundant, so 193 articles remained. Subsequently, after the application of the inclusion and exclusion criteria, 114 articles were removed, leaving 79. Of these, 6 articles were not accessible in full, leaving 73. Finally, other criteria, such as a lack of relevant data or not being a fit for this review, led to the elimination of 8 more articles, and thus a total of 65 articles were included in this study. The information was extracted from the selected articles, processed, and grouped by association. A summary table (Table 1) was constructed to facilitate the reading of the articles.

### 3.2. Most-Used Photosensitizers

**Figure 2:**
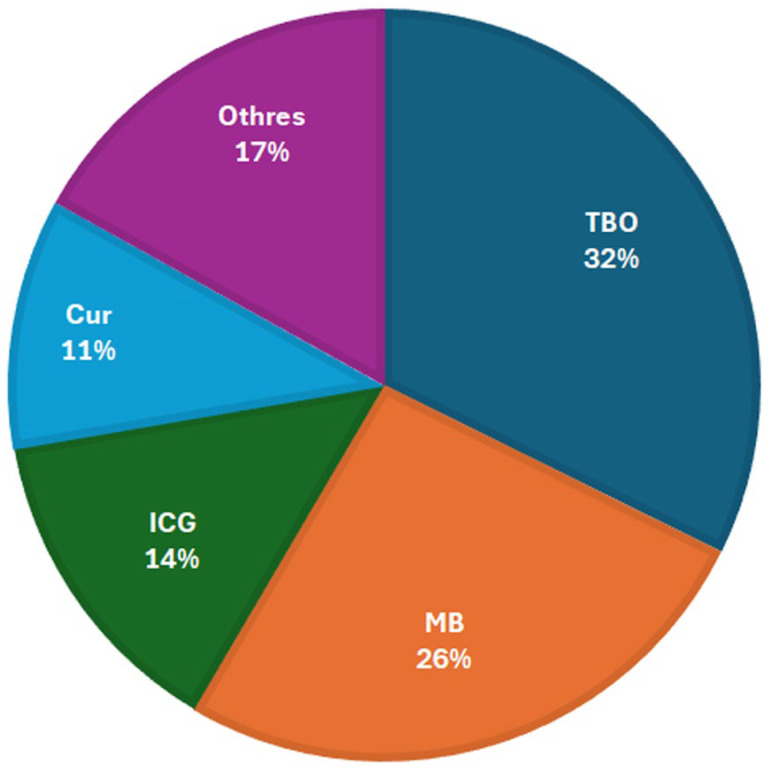
The proportions of the most-used photosensitizers against *Enterococcus* spp. found in this work. TBO: toluidine blue, MB: methylene blue, ICG: indocyanine green, Cur: curcumin, and **Other**: less-represented PSs such as erythrosine or riboflavin.

To be appropriate for clinical use, a photosensitizer should be a pure compound that allows reproducibility in its manufacture. It must be non-toxic and, ideally, soluble in water to facilitate its distribution and elimination from the body. It should be antibacterial when irradiated and not exhibit obscure toxicity against mammalian cells. Another desirable property is a cationic charge that facilitates its proximity to the bacterial envelope [31]. To have a practical antibacterial effect, the Atlanta CDC and CLSI suggest that a PS must reduce the bacterial load by >99.9%, equivalent to 3 log_10_ [3,32]. Table 1 summarizes the photosensitizers that present antimicrobial activity against *Enterococcus* spp. of the 65 articles included in this review, The PSs found showed a reduction in bacterial load from 0 to 6.5 log_10_, and the main photosensitizers were toluidine blue (**TBO**) 21, methylene blue (**MB**) 17, indocyanine green (ICG) 9, and curcumin (Cur) 7 (Figure 2). Other works also used various formulations like FL., RB, HYP, HP, and nanoparticles (made of selenium, AgNP, etc.).

#### 3.2.1. Photosensitizers Based on TBO and MB

Antimicrobial applications of phenothiazine derivatives, including TBO and MB, have been widely studied for photodynamic action since the 1930s. These compounds show similar maximum absorption in the red spectrum (590–680 nm), significantly penetrating tissues [33]. Toluidine blue is one of the most studied PSs in antimicrobial photodynamic therapy, largely with regard to its application to biofilm-forming bacteria such as *Enterococcus* spp. TBO is a cationic and amphiphilic compound with a low molecular weight, and it is active against Gram-positive and Gram-negative bacteria [34]. Its action, both individually and in association with other compounds such as potassium iodide (**PI**), EDTA, and nanoparticles, has been widely studied [35,36,37].

As little as 10 µM of TBO has shown a bactericidal effect on *Enterococcus faecalis* biofilms by reducing bacterial load by 3.8 log_10_ or 5.5 log_10_ when irradiated with 5 J/cm^2^ or 10 J/cm^2^, respectively [38]. The dose dependence of the decrease in bacterial load is consistent with the observed increase in ROS production. The authors in question observed that the production of intracellular ROS also depends on the irradiation power used, which showed an increase of 7 and 12 times, respectively [38]. The light sources used to activate TBO have also been evaluated, comparing the efficacy of diode lasers with that of light-emitting diodes (LEDs) with respect to disinfecting a root canal [39]. For example, LED light at 635 nm/2000–4000 mW/cm^2^ was significantly better than diode laser light at 635 nm 3000 mW/cm^2^, even at the same power level, i.e., 90 J/cm^2^ [39]. On the other hand, the efficacy of PDT with TBO has been compared with the standard root-cleaning and disinfection method using 2.5% sodium hypochlorite (NaOCl) [32]. The authors concluded that combining NaOCl and PDT with 0.5 mg/L of TBO using a 625 nm diode laser significantly increased the disinfection efficiency for *E. faecalis* from 99.97% to 99.99% [40]. Similar results were shown when NaOCl and PDT were combined with other photosensitizers, such as MB and curcumin (Cur) [40]. On the other hand, PDT with TBO or MB does not show significant differences when comparing its bactericidal activity [41,42]. For example, using different light sources, namely, LEDs and a diode laser, López-Jiménez et al. (2015) [42] and Misba et al. (2017) [41] showed that TBO and MB inactivated the biofilm formation of *E. faecalis* with similar extents. Misba et al. used a concentration of 10 µM of each PS, and López-Jiménez et al. used a concentration of 0.1 mg/mL of TBO and 0.05 mg/mL of MB.

The second most relevant photosensitizer used to inactivate *Enterococcus* spp. is **MB** dye. MB is a non-toxic dye with a high quantum yield that generates singlet oxygen. It has an absorbance range in the red region at 590–660 nm, acting against Gram-positive and Gram-negative bacteria. For example, the PDT activity of MB has been demonstrated on both planktonic and biofilm-forming cells of *Enterococcus* spp. in endodontic treatments [43]. To optimize the elimination of *E. faecalis* in bacterial suspensions, Soares et al. 2018 [44] evaluated multiple variables such as energy dose, renewal of the photosensitizer, and application cycles. The authors verified that light activation, when applied in cycles that allow PS renewal, increases the effectiveness compared to applying the entire exposure dose at once [44]. Furthermore, the effectiveness increases depending on the increase in the dose of accumulated energy. Several authors [44,45,46,47] evaluated the efficacy of PDT and MB combined with other antimicrobial disinfection methods. Similar to TBO, incorporating MB-PDT significantly improves the effectiveness of classic treatment with NaOCl alone [46] or combined with EDTA [45] for root canal cleaning. In one study, de Freitas et al. showed that combining MB-PDT with the antimicrobial peptide Aurein 1.2 improves the inhibition of *E. faecalis* biofilm formation [47]. This amphipathic cationic oligopeptide is found naturally in prokaryotic and eukaryotic cells and may destroy these cells in minutes, using the cytoplasmic membrane as their primary target. The synergistic effect with MB-PDT may be produced because Aurein 1.2 can modify the bacterial membrane’s permeability [47].

In the studies by Sampaio et al. (2020) and De Freitas et al. (2018), it was also observed that the efficiency of PDT depends on both the irradiation mode and the PS used when comparing the effectiveness of three photosensitizers: methylene blue (MB), curcumin (CUR), and Chlorine-e6 (Ce6) [48,49]. They probed variables such as PS concentration and continuous light irradiation vs. fractional light irradiation (on for 60 s and off for 60 s) [48,49]. The authors demonstrated that MB at 156 µM and Ce6 at 84 µM are more efficient when irradiated in fractional mode with 80 J/cm^2^ and 45 J/cm^2^ of energy, respectively, achieving the complete inactivation of *Enterococcus faecalis*, compared with continuous mode [49]. Continuous irradiation did not achieve complete bacterial elimination at any Ce6 concentration, but this result was obtained at a high light dose and PS concentration (100 J/cm^2^/157 µM) of MB. On the other hand, CUR showed better results for complete inactivation in continuous light irradiation mode (12.5 J/cm^2^), with a minor concentration of 34 µM applied to *Enterococcus faecalis* [49]. The authors observed that the synergistic effect depends on the PS used since it enhanced the PDT mediated by MB and Chlorine-e6 but not by curcumin. Therefore, it is interesting to evaluate different combinations of PSs and cationic antimicrobial peptides (AMP) [48] since their synergy allows the elimination of bacteria with minimum concentrations of both and low doses of light, which can minimize the adverse effects on the host tissues [48,49].

#### 3.2.2. Other Photosensitizers

**Curcumin** is a moderately water-insoluble phenolic compound that can be obtained naturally from the rhizomes of Curcuma longa or synthetically. Its absorption spectrum presents a band mainly at 430 nm, although it has a broader range in the blue region between 300 and 500 nm [50]. The rapid degradation rate of curcumin facilitates the treatment of local infections, preventing photosensitivity after a few hours, which gives it an advantage [51]. Using a light-emitting diode (LED), Rocha et al. observed that the PDT activity of curcumin inhibited *E. faecalis* biofilm formation [52]. The authors selected curcumin because its light absorption range is in the blue zone and since it has been shown to have good antimicrobial activity without damaging mammalian cells. For example, PDT treatment at 450 nm with 1.5 g/L of curcumin and a 20.1 J/cm^2^ light dose resulted in a significant reduction in *E. faecalis* biofilms (1.92 log_10_) compared to that observed in the control group [52]. Curcumin can be used at low concentrations; for example, de Annunzio et al. noted that as little as 136 µM could completely eradicate *E. faecalis* in PDT with light doses of 12.5 J/cm^2^ [51]. *E. faecalis* is characterized by resisting the disinfection process of endodontic treatment. Chelators such as EDTA and HEBP are widely used in endodontic practice as they facilitate the dissolution of the inorganic portion of the dental tartar layer. The combined activity of curcumin and the EDTA and HEBP chelators has been shown to be a valuable alternative for achieving complete bacterial eradication in dental plaque [53]. The combination of curcumin and chelators effectively reduced the viability of *E. faecalis* in an ex vivo model. The authors used bovine incisors contaminated with *E. faecalis* and exposed them to an energy dose of 75 J/cm^2^, resulting in a decrease in viability of 1.89 log_10_ for CUR-EDTA and 2.16 log_10_ for CUR-HEBP compared to that mediated by CUR alone (0.72 log_10_) [51,53].

Various authors have investigated the effectiveness of indocyanine green (**ICG**) dye as a PS [39,54,55,56,57,58]. ICG is a non-toxic, anionic cyanine dye approved by the Food and Drug Administration (FDA) for use in fluorescence angiography. It is activated by infrared light in the 800–810 nm spectrum, allowing greater tissue penetration. Therefore, its study in regard to PDT has emerged in the endodontic area as a possible alternative for applications for root canal disinfection. Other authors compared the disinfection capacity of root canals, namely, the “Gold standard” method of using **NaOCl** and Chlorhexidine (**CHX**), in combination with PDT-ICG activity [56,59]. The results showed effective disinfection of the root canal system using 100 µg/mL of ICG in PDT with a 5 W near-infrared (NIR) diode laser. However, the combination of ICG-PDT with pretreatment with 2.5% NaOCl showed no significant differences in *E. faecalis* eradication [59]. On the other hand, ex vivo studies that compared the effects of PDT-ICG, CHX at 2.0%, and the combination PDT-ICG+CHX at 2.0% demonstrated greater antimicrobial efficacy, with a 99.9% reduction compared to the control [56]. When comparing the effectiveness of ICG with that of the photosensitizers MB and TBO, the authors observed no significant differences in the reduction in *E. faecalis* burden. However, in this study, the authors determined the effect of sublethal doses of PDT with each PS on the expression of the esp gene associated with biofilm formation. They observed that treatment with ICG inhibited expression more significantly, which is why the authors attributed the advantage to inhibiting biofilm formation to a greater extent. PDT with MB, TBO, and ICG reduced the *esp* gene expression in *E. faecalis* by 2.8, 3.2, and 5.2 times, respectively [60].

During our search, we found other PSs whose usefulness for eradicating *Enterococcus* spp. has been less studied, such as **erythrosine**, **ALA-D**, rose Bengal (**RB**), fullerene, and hyperacin (HYP) [8,61,62,63,64,65]. Among them, the one that seems to show the most promising results against *Enterococcus* spp. is hypericin (**HYP**), a natural photosensitizer that stands out for being considered one of the next-generation drugs for PDT [66]. Hyperacin is a derivative of phenanthroperylenequinone with various medical applications, including as medication for depression [67] and wound healing [68]. Due to its absorption spectrum with maximums between 540 and 600 nm, it promises to be a helpful PS, reducing the viability of *E. faecalis* by 6.5 log_10_ and posing low cytotoxicity to mammalian fibroblasts [63].

### 3.3. Photodynamic Therapy in Clinical Isolates

Most studies of new antimicrobial drugs use standard laboratory strains such as ATCC, allowing better reproducibility of the results. Subsequently, to improve the knowledge of the behavior of these drugs, it becomes desirable to address strains that present more significant genetic variability, such as clinical isolates. For example, in 2015 and 2020, Tennert et al. evaluated the dental application of PDT in an in vivo and ex vivo study of 307 root canals infected with the clinical isolate of *E. faecalis* T9 [36,37]. The authors used different PSs and combinations, such as TBO + 90% isopropanol (IPA)-based photosensitizer and TBO + EDTA, or the synergy between NaOCl + PDT. The results showed that IPA-based photosensitizers significantly enhanced the antimicrobial effect of PDT, which was effective against the clinal isolate [36,37]. Other clinical isolates, such as *E. faecium* EU87 and *E. faecalis* EU92, were used to evaluate the synergistic ability of PDT and antibiotics to reduce the viability of biofilm formation. The authors showed reductions of 5 log_10_ and 2.5 log_10_ of CFU/mL using Fullene (0.5 μM) and Rose Bengal (0.1 μM), respectively [8].

### 3.4. Photodynamic Therapy in Ex Vivo and In Vivo Studies

A good approximation of the in vivo efficacy of photodynamic therapy is ex vivo experiments, which are relatively easy to perform on extracted teeth in dental medicine. Because *Enterococcus* spp. is a persistent microorganism in periapical infections, present in up to 77% of failed root canal treatments, many researchers are exploring its eradication in ex vivo experiments [37]. In preparation for ex vivo analysis, human teeth typically must be extracted for clinical reasons, generally without a history of caries, cracks, fractures, calcification, or restoration. Later, the teeth are disinfected with sodium hypochlorite (NaOCl) and decoronated using a diamond disc to access the root canal [69].

Biofilm reduction is one of the tests that can be easily replicated ex vivo for decoronated teeth. To this end, the prepared teeth are typically exposed to a culture of *Enterococcus* spp. and incubated for variable periods to allow biofilm formation before PDT treatment. For example, the ability of graphene oxide curcumin (**rGO-Cur**) to prevent the formation of *Enterococcus faecalis* biofilms was evaluated ex vivo in root canals and compared to the traditional NaOCl method [69]. The results showed that the minimum biofilm inhibitory concentration (MBIC) of rGO-Cur is 250 µg/mL and produces eight times more ROS. Furthermore, in a checkerboard assay, the MBIC value of rGO-Cur-PDT was reduced noticeably compared to the MBIC values of the controls with no light (rGO-Cur) or no PS (LED irradiation solely) applied for *E. faecalis* [69]. For example, complete eradication of *E. faecalis* was achieved when the MIC was reduced to 1/8 but at irradiation times of 240–300 s or using half the MIC for 60 s [69]. Other researchers tested ex vivo in human teeth the usefulness of ultrasonic activation [70] or the single-file technique using an instrument to prepare a root canal to improve the ability of MB-PDT to eradicate *Enterococcus* spp. [46]. For ultrasonic activation, the MB compound was irrigated using an intracanal syringe since this improves the action of the introduced substances. Ultrasonic activation provides higher adhesion and better distribution of the MB in the root canal in all areas of the colonized space, significantly improving the effectiveness of PDT [70]. On the other hand, it demonstrated that the application of a single filing in combination with 2.5% NaOCl and PDT-MB is the most effective treatment for root canal disinfection [46]. Both researchers concluded that the ex vivo model provides significant information on the effect of PDT and agree that this model was essential for evaluating the antimicrobial efficacy of the disinfection methods associated with PDT.

In vivo studies allow researchers to verify the effectiveness of PDT in reducing bacterial loads in a natural context. Our search found only two published studies in which PDT was used in vivo to treat *Enterococcus* spp. colonization/infection. For example, a randomized clinical study involving 39 patients showed that PDT and a high-power diode laser (DL) could be used as adjuvants in the standard endodontic treatment of chronic periapical periodontitis (CPP) affecting young permanent teeth [71]. The participants were randomly divided into three groups (PDT, DL, and a control), and bacterial identification and quantification were performed using MALDI–TOF spectrometry. The control group was treated with the regular endodontic disinfection procedure, and although it significantly reduced the bacterial load, it did not result in complete eradication. In comparison, PDT and DL, used as adjuvants, successfully eradicated eight and six bacterial genera/species, respectively [71]. On the other hand, six healthy volunteers with no signs of gingivitis or caries were recruited to evaluate PDT’s capacity to eradicate single species of planktonic bacteria and microorganisms during initial oral bacterial colonization in situ. Using visible light and water-filtered infrared-A (**VIS+wIRA**) to activate TBO, the researchers reduced *Streptococcus mutans* and *E. faecalis* colony numbers by 2 log_10_ via PDT [72].

### 3.5. PDT Combined with other Therapies/Drugs

Photosensitizers are often combined with other compounds to improve their photodynamic activity, and we searched for information on whether mixtures had been used for PDT against *Enterococcus* spp. Although combining photosensitizers with antibiotics to treat multiple bacteria is widely studied, our search only found one work that tested it against Enterococcus. In said study, the PSs rose Bengal (**RB**) and some fullerene derivatives (FL) were tested in PDT combined with a set of ten antibiotics recommended by the European Committee of Antimicrobial Susceptibility Testing (EUCAST) for the inactivation of clinical isolates of *E. faecalis* and *E. faecium* [8]. Although the results varied depending on the strain and the method used, it can be highlighted that PDT-RB was synergic with gentamicin and ciprofloxacin against *E. faecium* but antagonistic with vancomycin and daptomycin. On the other hand, PDT-RB was synergistic with gentamicin, ciprofloxacin, and daptomycin against *E. faecalis*. *E. faecalis* was also sensitive to PDT-FL working synergistically with imipenem [8]. These and other authors suggest that the synergy observed is partially due to the permeabilization of the bacterial envelope; however, the exact mechanism is still unknown [8,73]. Therefore, the results show that combined treatment can be a great alternative for combatting multidrug-resistant *Enterococcus* spp.

Other compounds that can improve the activity of a PS include chelators and antimicrobial peptides [47]. For example, the aurein 1.2 peptide increased the PDT activity of TBO and chlorin-e6 and was a feasible alternative for eliminating *Enterococcus faecalis* by increasing the internalization of a PS in the bacterial cytoplasm [48]. On the other hand, the chelator EDTA can increase the bactericidal activity of PSs by permeabilizing the bacterial plasma membrane and helping to dissolve the inorganic matter left from manual dental disinfection, improving the penetration of a PS into the root canal [36,53,74]. However, PDT has proven to be an excellent alternative for eradicating *Enterococcus* spp. infections in root canals; several authors have indicated that it is an ineffective disinfectant when used alone. However, as a complementary treatment, it significantly improves the classic chemomechanical treatment, eliminating the bacterial load in most studies [40,59,71,75].

### 3.6. PDT Associated with Nanoparticles

Nanoparticles are submicroscopic compounds that range from 1 to 100 nanometers in size and whose application has significantly increased in photodynamic therapy in recent years since they help with some limitations that photosensitizers present [76]. For example, functioning as a carrier, integrating nanoparticles into a PS can improve the PS’s distribution to the target cells [77] and prevent its premature release, i.e., release before reaching the target [78]. Finally, NPs can be modified by adding functional groups that provide specificity in therapy [79,80]. Four types of NP/PS interaction have been described as being applicable during photodynamic therapy: (1) photosensitizers embedded in nanoparticles, (2) photosensitizers attached to the surfaces of the nanoparticles, (3) photosensitizers on one side of the nanoparticles, and (4) nanoparticles used as photosensitizers [81]. One way to present nanoparticles is by including them in support structures such as acrylic dentures. For example, graphene and silver nanoparticles synthesized using the radiofrequency-catalyzed chemical deposition (RF-CCVD) method were added as a powder at 1% and 2% to a commercial acrylic resin and allowed the formation of discs with a diameter of 5 mm and a thickness of 1 mm. The antimicrobial activity of these discal structures was proved using the Kirby–Bauer method in Mueller–Hinton plates inoculated with *Enterococcus faecalis* [82]. The authors used a laser and a light-emitting diode to excite the NPs in the acrylic disc, and inhibition zones were observed, showing significant differences for the light-treated samples. The authors concluded that extraoral photodynamic therapy applied to denture resins exhibits improved antibacterial activity. [82]. To improve the photodynamic activity of MB, other authors combined it with selenite nanoparticles (**SeNPs**) through chemical reduction. Using a 630 nm LED light with an output power of 200 mW/cm^2^, the authors observed an increased photodynamic activity of the MB/SeNP combination [61]. They also tested the degree to which the photodynamic activity of toluidine blue (TBO) improved when combined with silver nanoparticles (**AgNPs**) [83]. TBO/AgNPs were used to treat infections with planktonic *E. faecalis* in the root of a dental canal. The studies verified the most appropriate proportion was 20 ppm of TBO + 10 ppm of AgNPs, activated with a FotoSan^®^ LED light (CMS Dental, Copenhagen, Denmark). The bacterial inactivation was between 99.7% and 99.9% in biofilms 21 days old. The authors observed decreases in *E. faecalis* load of 2.14 log_10_ and 2.7 log_10,_ respectively, in extracted teeth [83]. On the other hand, rose bengal was functionalized with chitosan nanoparticles to improve its photodynamic activity. Rose-bengal-functionalized chitosan nanoparticles (**CSRBnp)** particles were synthesized using chemical cross-links of carbodiimide [N-ethyl-N=-(3-dimethylamino propyl). The photodynamic activity of CSRBnp was tested with *E. faecalis* ATCC 29212, with doses of 5–10 J/cm^2^, and showed 100% effectiveness even in the presence of the pulp and BSA inhibitors [84]. Another way to improve photodynamic activity is by using polymer nanoparticles, which, for example, can be coupled with natural photosensitizers such as curcumin. The synthesis of a novel polymeric nanoparticle of poly (lactic-co-glycolic acid) (**PLGA**) loaded with curcumin (**PLGA+Cur**) increased the curcumin photodynamic activity for *E. faecalis* in dentin tubules of bovine teeth [15]. Blue-light-emitting diode (LED) irradiation was applied at a 72.6 J/cm^2^ fluency to reduce the curcumin MIC from 500 µg/mL to 100 µg/mL against planktonic cultures of *E. faecalis*. Also, the use of NP+Cur at 325 and 200 µg/mL resulted in 2.06 and 1.83 log10 reductions in *E. faecalis* biofilm compared to the control group. Interestingly, the authors tested the therapy using 325 µg/mL of NP+Cur photoactivated in a biofilm formed by three bacterial species, namely, Streptococcus oralis, Actinomyces viscosus, and *E. faecalis*, mimicking conditions found in the oral biota and resulting in a reduction of 5.08 log_10_ compared to the control group [15].

### 3.7. Synergism of PDT with Antibiotics and Antiseptics

In our review, we have already shown that different photosensitizers can be combined to improve the outcome of phototherapy. However, in this section, we wanted to focus primarily on synergism with antibiotics, which is one of the most used strategies to treat infections and, at the same time, can prevent the appearance of antibiotic resistance. Although our study only found one trial evaluating possible synergism, the results are promising. The authors found that photodynamics positively affected susceptibility to several antibiotics recommended by EUCAST for treating *Enterococcus* spp. [8]. In sublethal aPDI conditions, the combination of RB and fullerene (FL) with 10 antibiotics to inactivate planktonic and biofilm formation increased susceptibility to numerous antimicrobials, showing larger inhibition zones and decreasing the MICs. For example, the inhibition zone of doxycycline increased from 9.4 to 11.3 mm, and the MIC value for gentamicin decreased from 6 to 3 µg/mL. The authors saw a post-antibiotic effect where the PDT treatment delayed the growth of *Enterococcus faecium* clinical isolates when combined with gentamicin, streptomycin, tigecycline, doxycycline, and daptomycin [8]. Consequently, the PDT was effective in sensitizing bacteria to resistant antibiotics. Moreover, checkerboard FICI calculation for *E. faecalis* confirmed synergy with values of <0.5 for the antibiotics gentamicin (0.38), ciprofloxacin (0.38), and daptomycin (0.16). However, it showed no synergy with values >0.5 for streptomycin, tigecycline, doxycycline, linezolid, imipenem, vancomycin, or ampicillin antibiotics [8].

We have seen that the combination of photosensitizers with frequently used antiseptics such as NaCLO increases the effectiveness of the treatment as a whole. Other authors tested disinfectant compounds, such as the cationic antiseptics benzalkonium chloride (**BAC**) and chlorhexidine digluconate (CHX) combined with photosensitizers such as sulfonate methyl pyridinium (**TMPyP)**. The authors demonstrated that PDT allows sublethal concentrations of BAC and CHX to inactivate *E. faecalis* completely. Individual concentrations decreased by 50% for TMPyP, 23–40% for BAC, and 18–43% for CHX [85]. Furthermore, the combined application reduces the concentrations of both the PS and the adjuvant compared to the application of each separately.

**Table 1:** List of studies and the photosensitizers used in developing PDT for *Enterococcus* spp.

Bacteria	PS/Dose	PDT/Dose	Study/Reduction	Applications	Refs.
*Enterococcus faecium*	TBO/16 µg/mL, in IPA at 90%	LED 635 nm/100 mW	Ex vivo/Bacterial reduction 99.3%	Improving PDT efficiency in root canals ex vivo	[37]
*Enterococcus faecalis* IBRC-M 11130	SIOXYL + Solution (3% HP in water solution stabilized with glycerophosphate).	Diode laser; 980 nm/320 μM, 2.5 W	In vitro/Reduction of 2 log_10_ (*p* < 0.05)	Planktonic and biofilm-producing bacteria.	[86]
*Enterococcus faecalis* ATCC 29212	- AgNPs (10 ppm) - TBO (0.02 mg/mL) (20 ppm) - Mix of 20 ppm of TBO + 10 ppm of AgNPs	LED (FotoSan); 620–640 nm/2.000–4.000 mW/cm^2^.	Ex vivo/Reduction of 2.7 log_10_	Treatment of infected root canals.	[83]
*Enterococcus faecalis*	TBO/15 mg/mL Diluted in 0.9% NaCl.	LED; 635 nm/100 mW	Ex vivo/Reduction of 99.9%	Ultrasonic activation and chemical Modification of PDT against root-canal infections	[36]
*Enterococcus faecalis* ATCC 29212 *Enterococcus faecium* VRE ATCC 700221	MB; c-E6; (Cur). Aurein 1.2 (UA)	450 nm (155 mW/cm²); methylen blue and chlorine-e6 ligth at 660 nm (151 mW/cm²).	In vitro/17, 34 and 68 μM Cur/reduction 5.2%; 21, 42, and 84 μM chlorine-e6 (Ce6)/reduction 10.3%; 39, 78, and 156 μM UA/reduction 3.45%; 16 μM, 16% and 32% reduction, respectively.	Planktonic and biofilm-producing bacteria.	[48]
*Enterococcus faecalis* ATCC BAA-2128	Pyoktanin blue (PB)/1% (200 μL)	Diode laser; 808 nm/3 W, 60 s	In vitro/Reduction of 100%	Planktonic and biofilm-producing bacteria.	[87]
*Enterococcus faecium* EU87 *Enterococcus faecalis* EU92	RB 0.1 µM, FL 0.5 µM	LED; 522 nm/ 6.4 J/cm ^2^	In vitro/Reduction of 2.5 log_10_ (RB); 5 log_10_ (FL).	Planktonic and biofilm-producing bacteria.	[8]
*Enterococcus faecalis*	TBO + 17% EDTA	Red laser; 660 nm/6 J/cm^2^ at 100 mW.	Ex vivo/Reduction of 97.6%	Treatment of infected root canals. Final irrigation in root canals of the primary teeth.	[74]
*Enterococcus faecalis* (T9)	TBO 0.9%/at 5, 10, 25 and 50 mg/mL	VIS+wIRA white light and infrared 580–1400 nm/200 mW/cm^2^	In vivo/Reduction of 99.99%	Use of visible light plus water-filtered infrared-A (wIRA).	[72]
*Enterococcus faecalis*	MB/0.01%	Laser Diode; 660–690 nm/ 9 J/cm^2^ at 100 mW	Ex vivo/Reduction of 3.60 ± 0.19 log_10_	Ultrasonic activation of photodynamic therapy of root canal.	[70]
*Enterococcus faecalis*	ICG/100 µg/mL	Diode laser; (1 W)/ 286 J/cm^2^	Ex vivo/Disinfection amounting to 99.99% (*p* < 0.01)	Near-Infrared Diode Laser for Infected Root Canals.	[59]
*Enterococcus faecalis* ATCC 29212	Riboflavin (Rb)/0.01%	UVA; 365 nm/Irradiancy 3 mW/cm^2^, dose 5405 J/cm^2^	In vitro/Reduction of 60 to 70%.	Photodynamic UVA-riboflavin for bacterial elimination.	[88]
*Enterococcus faecalis*	MB (38; 78; 156; 234; 312 µM) Ce-6 (20; 42; 84; 126; 168 µM) Cur (16; 34; 68; 135 µM)	LED; 450–660 nm/ 12.5 to 120 J/cm²	In vitro MB: Complete reduction Ce-6: No reduction Cur: Complete reduction	Planktonic and biofilm-producing bacteria.	[49]
*Enterococcus faecalis* ATCC 29212	ALAD/5%	TL-01 (LED 630 nm/380 mJ/cm^2^) TL-03 (LED 630 nm/6 mJ/cm^2^)	In vitro/Reduction equal to 1.5 log_10_	Comparison between single and multi-LED emitters for application to planktonic and biofilm-producing bacteria.	[62]
*Enterococcus faecalis* ATCC 29212	MB/0.01% (31.2 mol/L) RB (25 mol/L)	MB: red laser 660 nm/40 mW RB: Green laser 532 nm/40 mW	In vitro/Rb reduction to 0.12 log10^8^ from 2.82 log10^8^ UFC/mL. No reduction for MB.	Planktonic and biofilm-producing bacteria.	[89]
*Enterococcus faecium* ATCC 19434	Cur-PpIX/ 100 μg/mL.	LED; 405 nm/ 25.3 J/cm^2^ (84.5 mW/cm^2^)	In vitro/Complete reduction	Planktonic and biofilm-producing bacteria.	[90]
*Enterococcus faecalis* ATCC 9854	TBO/0.1 mg/L	Diode laser; 810 nm/0.2 CW. LED 630 nm, 200 mW/cm^2^	Ex vivo/LED reduction 4.88 ± 0.82 Laser reduction of 5.49 ± 0.71	Comparison of the antibacterial activity of diode laser and LED lamp in extracted human anterior teeth	[91]
*Enterococcus faecalis*	MB/1 mM RB/5 mM	Laser; red 660 nm and green 565 nm/60 mW/cm^2^	In vitro/MB Reduction of 86.50 ± 5.78%; RB reduction of 91.5%	Planktonic and biofilm-producing bacteria.	[92]
*Enterococcus faecalis* ATCC 19433	MB/0.005 μg/mL	Diode Laser/40 mW	In vitro/Reduction of 3.04 log_10_	Optimized elimination of planktonic and biofilm-producing bacteria.	[44]
*Enterococcus faecalis* ATCC 19433	Eritrosin/5 and 10 μM	LED; 440–480 nm/96 J/cm^2^	In vitro/5 uM Reduction of 5.517 log_10_ UFC 10 uM complete eradication	Planktonic bacteria.	[64]
*Enterococcus faecalis* ATCC 29212	TBO 0.1 mg/mL in NaCl at 0.9%	LED; 635 nm/2000–4000 mW/cm^2^ (90 J/cm^2^)	In vitro/TBO-LED Reduction of 1.60 × 10^4^ TBO-LASER Reduction of 2.26 × 10^4^	Laser vs LED for planktonic and biofilm-producing bacteria.	[39]
*Enterococcus faecalis*	MB/70 μL	Diode laser; 660 nm	In vitro/Reduction of Group 1 min 1.50 × 10^7^ Group 2 min 2.44 × 10^7^ Group 4 min 2.31 × 10^7^	Antimicrobial efficiency at different irradiation times for planktonic and biofilm-producing bacteria.	[93]
*Enterococcus faecalis* ATCC 11700	HYP/1 ug/mL	White LED with a yellow filter (590 nm)/30 mW (80 mW/cm^2^)	In vitro/Reduction of 6.5 log_10_. No significant cytotoxicity in fibroblasts	Fibroblast cell culture in bacterial suspension.	[63]
*Enterococcus faecalis* ATCC 2729	TBO/10 µM	Laser; (No-MRL-III) 630 nm/Group 1 5 J/cm^2^; Group 2 10 J/cm^2^	In vitro/reduction Group 1 3.8 log_10_; Group 2 5.5 log_10_	Planktonic and biofilm-producing bacteria.	[38]
*Enterococcus faecalis* ATCC 29212	Nanoparticle: chitosan (1%) and sodium selenite (Na_2_SeO_3_) 10 mM; (SeNPs) PS: MB 0.5 mg/mL	LED; 630 nm/200 mW/cm^2^	In vitro/MB+SeNPs showed a significant reduction in bacteria compared to untreated controls.	Planktonic and biofilm-producing bacteria.	[61]
*Enterococcus faecalis* ATCC 29212	Cur/1.5 g/L	Blue LED; 450 nm/67 mW/cm^2^, (20.1 J/cm^2^)	In vitro/Reduction: 1.92 log_10_ CFU/mL	Planktonic and biofilm-producing bacteria.	[52]
*Enterococcus faecalis* ATCC 29212	Cur/600 μmol/17% EDTA 18% HEBP Cur + EDTA 17% Cur + HEBP 18%	Blue LED; 455 ± 30 nm/40 mW/cm^2^	Ex vivo/Reduction Cur + EDTA – 1.89 log_10_. Cur + HEBP – 2.16 log_10_ Cur – 0.72 log_10_	PDT associated with different chelators against *Enterococcus faecalis* biofilms	[53]
*Enterococcus faecalis* ATCC 29212	PC-1/100 uM	LED; 735 nm/30 J/cm^2^	In vitro/Reduction 5.7 log_10_.	PC-1 in lipid vesicles with an optical concentration of *E. faecalis* was investigated.	[94]
*Enterococcus faecalis* ATCC 29212	Silver graphene nanoparticles at 1 and 2% with TBO, MB	Diode, red laser; 630 nm	In vitro/Reduction P2 = 0.22785 P1 = 0.38156	Planktonic biofilm-producing bacteria and control of halitosis-causing bacteria in denture wearers	[82]
*Enterococcus faecalis*	Phenothiazine chloride/10 mg/mL	High-power laser; 940 nm/1 W	Prospective clinical study/Complete elimination of *E. faecium*	Treatment of young permanent teeth with chronic periapical periodontitis	[71]
*Enterococcus faecalis* ATCC 29212	Chitosan (CSRBnp)/0.3 mg	Fiber-filtered light at 540 nm/5 J/cm^2^ × 1.66 min.	In vitro/Complete eradication *Enterococcus faecalis*	Treatment for reducing bacterial adhesion to the tooth dentin and the formation of root biofilms.	[84]
*Enterococcus faecalis* ATCC 29212	UCNP@SiO_2_/MB@QCh/2 mg/mL	Laser, NIR; 980 nm/1.5 W/cm^2^ × 10, 20 y 30 min.	In vitro/Eradication of over 99% of *E. faecalis*	The nanoparticles showed good biocompatibility and bactericidal activity, demonstrating safe clinical use.	[95]
*Enterococcus faecalis* ATCC 29212	- AgNPs (10 ppm) - TBO (0.02 mg/mL) - Mix of 20 ppm TBO + 10 ppm of AgNPs	Laser diode (FotoSan); 620–640 nm/2000–4000 mW/cm^2^.	Ex vivo/Reduction of 2.7 log_10_	Treatment of infected dental canal.	[83]
*Enterococcus faecalis* ATCC 29212	Nanoparticles: chitosan (1%) and Na_2_SeO_3_ 10 Mm; (SeNPs) PS: MB 0.5 mg/mL	Laser diode; 630 nm/200 mW/cm^2^	In vitro/MB+SeNP, a significant bacterial reduction.	Planktonic biofilm-producing bacteria.	[61]
*Enterococcus faecalis* ATCC 29212	Silver graphene nanoparticles at 1 and 2% with TBO and MB	Red laser diode at 630 nm	In vitro/Reduction P2 = 0.22785 P1 = 0.38156	To control halitosis and protect dental prostheses via planktonic and biofilm-producing bacteria eradication.	[82]
*Enterococcus faecalis* CIP 76.1170	THPP@AcLi/2.5 μM	White-diode laser/4.16 J/cm^2^	In vitro/99.999% bacterial removal	Water disinfection and other applications	[96]
*Enterococcus faecalis* ATCC 10.100	PLGA polymeric nanoparticles loaded with curcumin (NP+Cur)/325 μg/mL and 200 μg/mL	Blue-diode laser; 450 nm/72.6 J/cm^2^ (22 mW/cm^2^)	In vitro/Reduction of 7.90 log and 5.18 log, respectively	Elimination of biofilms of bacteria associated with dentin	[15]
*Enterococcus faecalis* ATCC 29212	Fe-MIL-88B-NH2: 1 mg/mL Al-MIL-101-NH2: 1 mg/mL Fe-MIL-101-NH2: 1 mg/mL ICG: 0.5 mg/mL	Laser diode; 810 nm/31.2 J/cm^2^	Ex vivo and in vitro/ Fe-88-ICG, Al-101-ICG, and Fe-101-ICG: 76%, 84%, and 89%, respectively (ex vivo) Fe-88-ICG, Al-101-ICG y Fe-101-ICG: 45.12%, 60.72%, and 62.67%, respectively (in vitro).	Good stability for use against biofilm formation and the growth of bacteria such as *E. faecalis* in vitro.	[97]

AgNPs: silver nanoparticles; ALAD: 5-delta-aminolevulinic acid; Cur: curcumin; FL: fullerene; HP: hydrogen peroxide; HYP: hyperacin; ICC: indocyanine blue; MB: methylene blue; Na_2_SeO_3_: sodium selenite; PB: pyoktanin blue; PC-1: zinc II phthalocyanine derivative; PLGA: poly (lactic-co-glycolic acid); PpIX: protoporphyrin; RB: rose bengal; Rb: rivoflavin; SeNP: selenium nanoparticles; TBO: toluidine blue; THPP@AcLi: 5,10,15,20-tetrakis(4-hydroxyphenyl)-21H,23H-porphyrin -loaded lignin nanoparticles; TMPyP: 5,10,15,20-Tetrakis(1-methyl-4-pyridinio)porphyrin tetra(*p*-toluenesulfonate).

## 4. Discussion

Multidrug resistance to antibiotics is a severe public health problem responsible for almost 700 thousand deaths yearly [4]. One of the main MDR bacteria is *Enterococcus* spp., which is responsible for causing HAIs, including urinary tract infections, and has a notable presence in persistent endodontic infections. Due to their multidrug resistance, the WHO classifies these bacteria within the priority group for the search for new antimicrobial therapies [1]. Photodynamic therapy (PDT) has proven to be a viable and effective complementary antimicrobial approach for treating multiple types of MDR bacteria [1,2]. PDT complements conventional antibiotics, antiseptics, and mechanical treatments in dental hygiene. Since the mechanism of action of PDT requires the simultaneous presence of the photosensitizer compound, molecular oxygen, and visible light, PDT can easily be applied to the oral cavity. Using red or blue LED light, PDT for periodontitis can effectively attenuate bacteria associated with periodontal diseases [98]. Dentists already have devices that emit light at specific wavelengths that can be easily applied to activate PSs on exposed oral cavity surfaces in the presence of oxygen [8]. Therefore, improving the effectiveness of PDT for *E. faecium* and *E. faecalis* will depend on multiple variables, such as the selected PS and its concentration, the wavelength at which the PS is activated, the power output of the lamp used, and the irradiation time, among others. For example, riboflavin or curcumin can be activated by using curing resin devices that emit light at wavelengths between 430 and 480 nm with powers close to 1200 mW/cm^2^ [98]. Thus, multiple PSs have been studied as treatment alternatives, with phenothiazine derivatives such as toluidine blue (TBO) and methylene blue (MB) being highlighted as the most effective. These phenothiazines have demonstrated high effectiveness in the treatment/elimination of various strains of *Enterococcus* spp. and other bacteria associated with dental diseases [15]. Photosensitizers characteristics, such as wavelength activation, make them suitable for PDT applications in multiple exposed areas or organs [38,39,44,57,99]. In endodontic applications, PDT also stands out for its capacity as an adjuvant to antiseptics such as NaOCl [85] or nanoparticles [83] in the total eradication of planktonic and biofilms of *Enterococcus* spp. [27,45,70]. Opposite to the good results yield by phenothiazine derivatives, curcumin did not show significant photosensitizer activity, even in combination with TBO or MB. Another drawback of curcumin is that its low solubility in water limits its elimination [51,52], although its low wavelength maximum absorption, close to 405 nm, makes it suitable for blue-light-emitting dental devices [98].

The light source used is another relevant parameter, and its selection will largely depend on the PS used, but its cost must also be considered. Thus, Afkhami et al. in 2020 and Asnaashari et al. in 2016 compared the antibacterial effects of TBO-mediated PDT based on two light sources: a diode laser and an LED. Since no significant differences were observed at the same power, LED lights were advised to be used because they cost less. Also, using high- or low-power lasers for prolonged periods can raise tissue temperatures, potentially causing thermal damage [39,91]. Along with the light source, the irradiation time required to obtain significant results must also be considered. Several authors propose to optimize it through fractionated irradiation, repeatedly exposing the tissue to the light source for short times, reducing the risk of tissue damage that could be caused by long periods of exposure. The effectiveness of this procedure will depend on the PS used; for example, MB increases its bactericidal efficiency, but not Cur, which maintains it [49].

Combining several photosensitizers with other compounds significantly improved the effectiveness of photodynamic therapy. For example, the results of synergy with antibiotics are promising as the authors demonstrated that it reverses the resistance of the strains, allowing the use of less-specific antibiotics in the treatment of MDR clinical isolates [8]. This synergy may be because PDT has multiple targets in the bacterial cell envelope, which can increase membrane permeability to antibiotics. On the other hand, the combined application of chelators, peptides, or chemical disinfectants is still being studied, yielding diverse results. However, some of these combinations have been shown to be very effective in eradicating *Enterococcus* spp. For example, mixes of Cur-EDTA, TBO-AMP, or MB-AMP could improve the delivery of PS or its uptake by the bacterial cell. The small number of studies carried out in vivo does not allow us to confirm the usefulness of PDT for treating infections caused by *Enterococcus* spp. This lack of information is partly caused by the preponderance of ex vivo studies, which are varied but limited to endodontics. Therefore, it is necessary to advance the evaluation of the effectiveness of this therapy in eradicating infections in organs or tissues of living organisms, both for dental hygiene and other infections such as urinary tract infections. Therefore, further clinical studies involving human patients and the development of studies using animal models will accelerate the determination of the safety and efficacy of this therapy, improving the chances of this therapy being applied to humans.

## 5. Conclusions

Our search showed that the field of endodontics is where the most significant utility has been found for photodynamic therapy against *Enterococcus* spp. The analyzed data suggest that PDT may effectively reduce the viability of *Enterococcus faecium* and *Enterococcus faecalis* in vitro and ex vivo. As PDT is effective against planktonic bacteria and prevents biofilm formation, it may be a beneficial complementary therapy for treating infections. The best-studied PSs include phenothiazine derivatives such as toluidine blue (TBO) and methylene blue (MB), with promising effects as PDT-dependent bactericides, alone or in combination. Most of these results were found in the preclinical area through in vitro and ex vivo studies, mainly in the endodontic area, so more studies are needed to determine PDT’s in vivo effectiveness. Therefore, since a large amount of evidence demonstrates the bactericidal ability of PDT in vitro, and there are only a few in vivo studies, we conclude that future research efforts should focus on demonstrating the safety and efficacy of these PSs in vivo in animal models.

## Data Availability

No new data were created in this study. Data sharing is not applicable to this article.
